# Supplementary material for: Assessment of TROP2, CEACAM5 and DLL3 in metastatic prostate cancer: Expression landscape and molecular correlates
Source: NPJ Precis Oncol. 2024 May 17;8:104. doi: 10.1038/s41698-024-00599-6 (PMC11101486; doi:10.1038/s41698-024-00599-6)
Supplement: Supplementary file 1 — Supplementary Data [file 41698_2024_599_MOESM1_ESM.pdf]

SUPPPLEMENTARY DATA

ASSESSMENT OF TROP2, CEACAM5 AND DLL3 IN METASTATIC PROSTATE CANCER: EXPRESSION LANDSCAPE AND MOLECULAR CORRELATES

Azra Ajkunic, Erolcan Sayar, Martine P. Roudier, Radhika A. Patel, Ilsa M. Coleman, Navonil De Sarkar, Brian Hanratty, Mohamed Adil, Jimmy Zhao, Samir Zaidi, Lawrence D. True, Jamie M. Sperger, Heather H. Cheng, Evan Y. Yu, Robert B. Montgomery, Jessica E. Hawley, Gavin Ha, Thomas Persse, Patricia Galipeau, John K. Lee, Stephanie A. Harmon, Eva Corey, Joshua M. Lang, Charles Sawyers, Colm Morrissey, Michael T. Schweizer, Roman Gulati, Peter S. Nelson, Michael C. Haffner

Supplementary Figure 1. *Ajkunic, Sayar et al.*

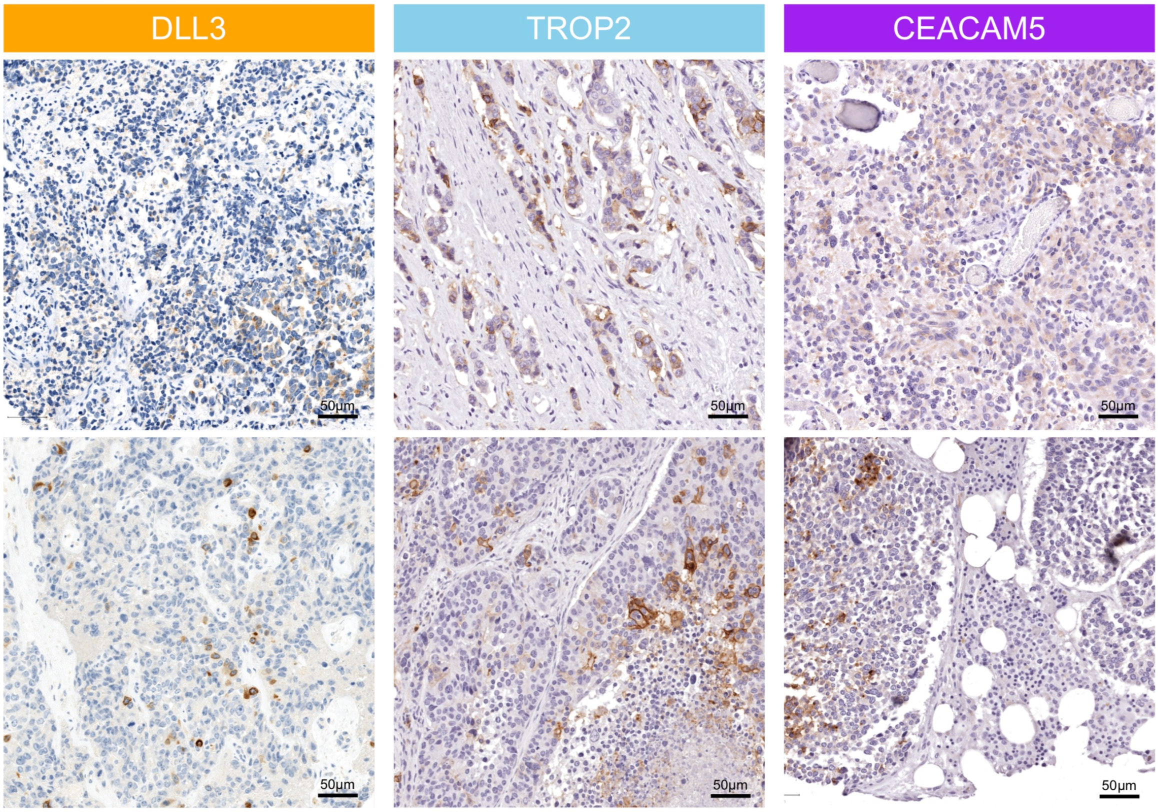

Supplementary Figure 1. Representative micrographs of mCRPC tumors with DLL3, TROP2 and CEACAM5 expression H-scores of 20.

Supplementary Figure 2. *Ajkunic, Sayar et al.*

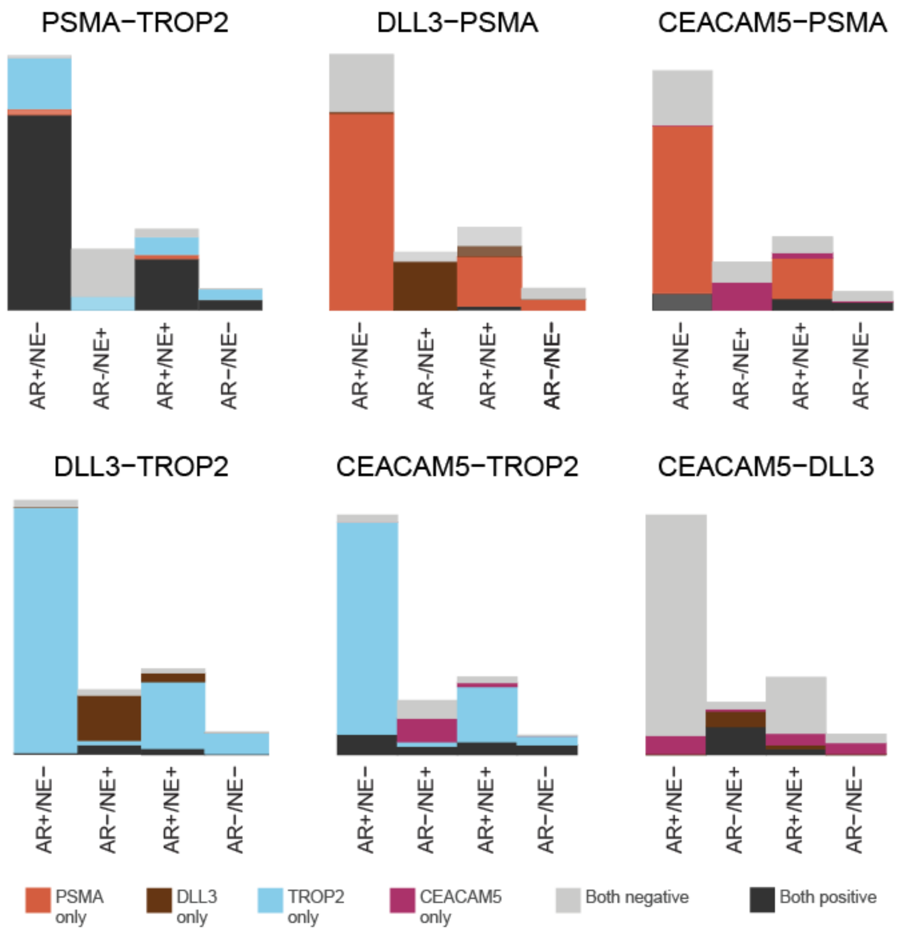

Supplementary Figure 2. Distribution of PSMA, TROP2, DLL3 and CEACAM5 co-expression pattern.

Supplementary Figure 3. *Ajkunic, Sayar et al.*

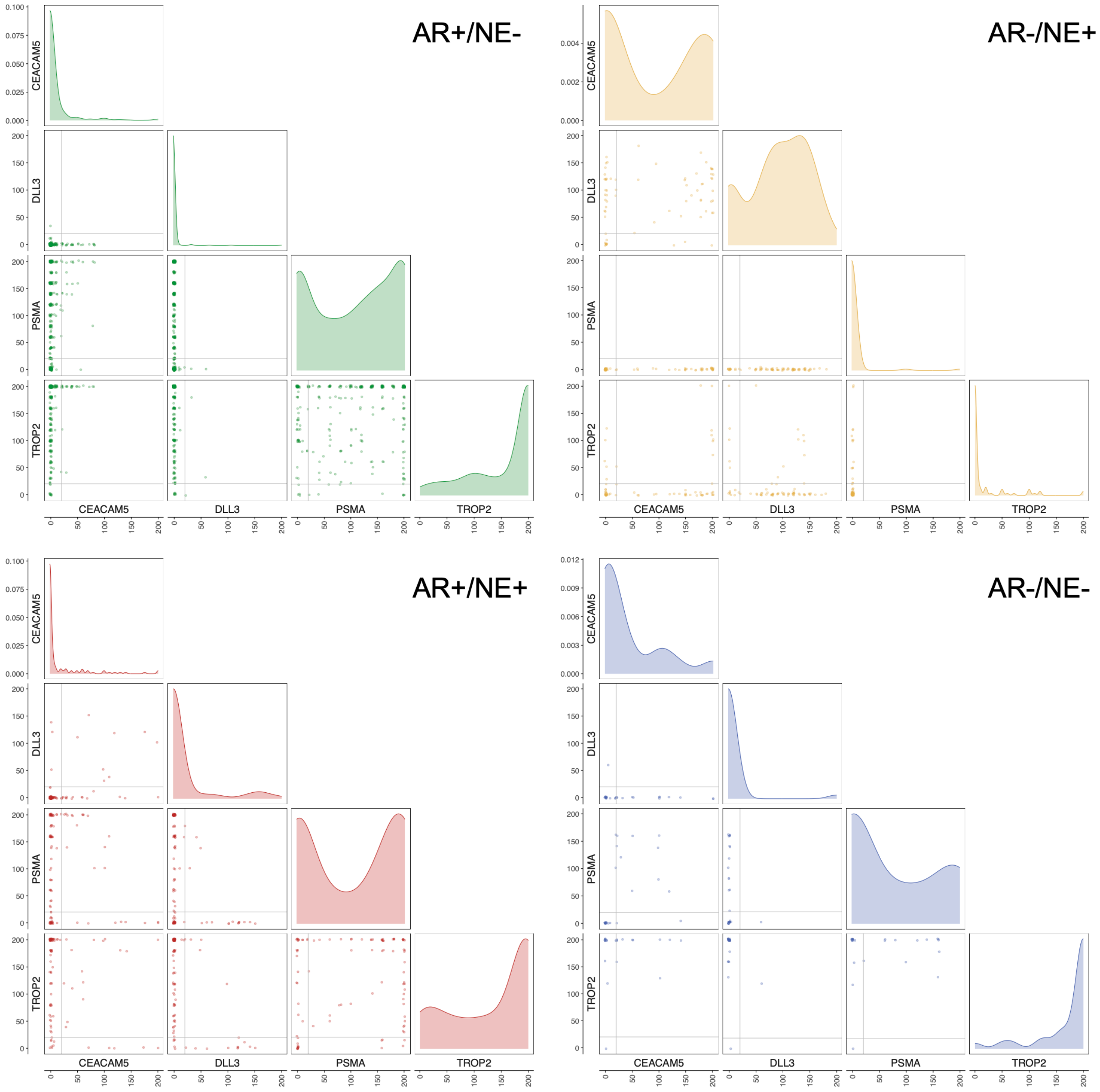

Supplementary Figure 3. Scatter and density plots show the distribution of co-expression patterns of CEACAM5, DLL3, PSMA and TROP2 across different molecular subtypes.

Supplementary Figure 4. *Ajkunic, Sayar et al.*

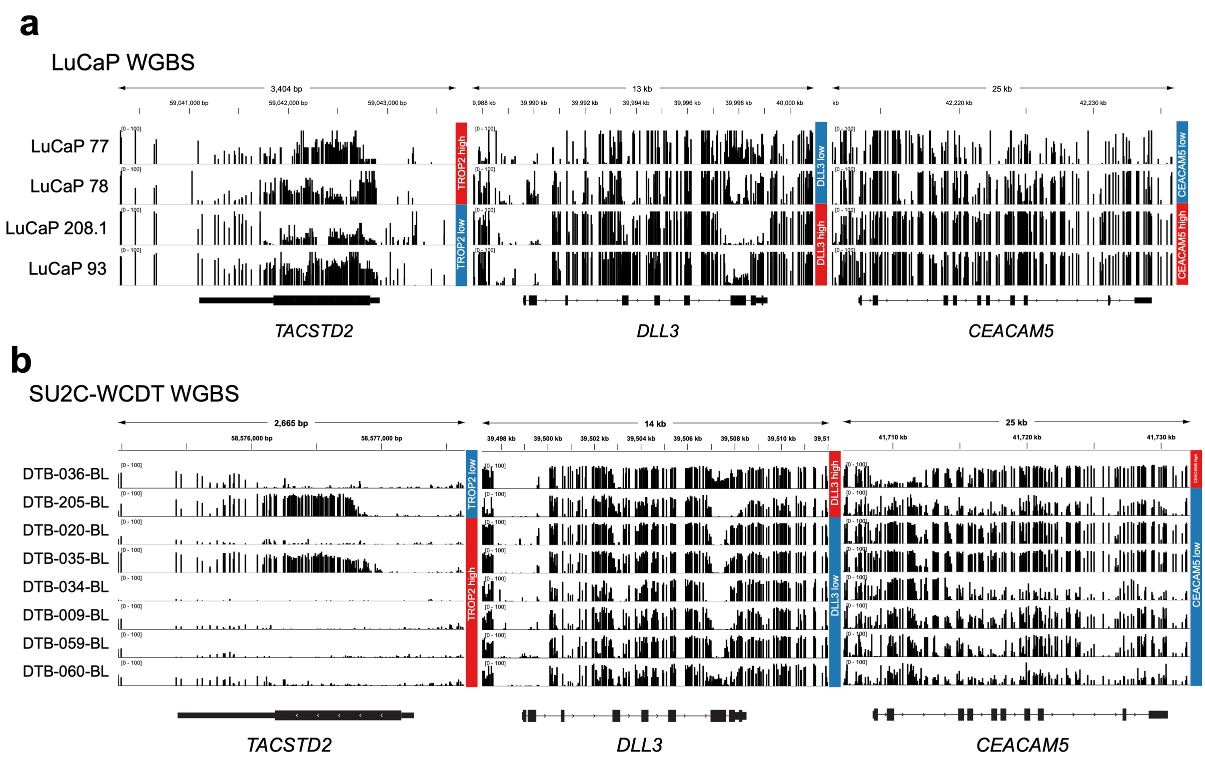

Supplementary Figure 4. Whole genome bisulfite sequencing studies show no consistent difference in CpG methylation pattern at the transcriptional start site of TACSTD2, DLL3 and CEACAM5 between high- and low-expressing tumors in **A.** LuCaP PDX lines and **B.** cases of the SU2C-WCDT cohort.

Supplementary Table 1. Clinical characteristics of patients from the UW-TAN rapid autopsy study with IHC expression data.

Total n = 52

**Demographics data**	**Median**	**Inter quartile range**
Age at initial diagnosis (median [IQR])	60.6	[53.92, 66.33]
PSA at diagnosis (median [IQR])	19.5	[7.55, 111.25]
Age at death (median [IQR])	68.6	[61.91, 74.43]
PSA at death (median [IQR])	90.6	[8.69, 1007.51]

**Treatment history**	**Number of patients**	**Percent**
Androgen deprivation therapy (ADT)	51	98.1
Abiraterone acetate	33	63.5
Enzalutamide	28	53.8
Taxane therapy	36	69.2
Carboplatin	27	51.9
Radiation therapy	36	69.2
Alpharadin	9	17.3

**Metastatic sites**	**Number of patients**	**Percent**
Bone	48	92.3
Liver	36	69.2
Lymph nodes	36	69.2
Lung	31	59.6
Adrenal	13	25.0
Spleen	7	13.5
Other soft tissue sites	18	34.6

Supplementary Table 2. Co-expression pattern of cell surface antigens across different molecular subtypes of CRPC.

PSMA-TROP2

subtype	expressed	percent
AR+/NE-	Both	76.6
AR+/NE-	PSMA only	2.3
AR+/NE-	TROP2 only	20.1
AR+/NE-	Neither	1.0
AR-/NE+	Both	0.0
AR-/NE+	PSMA only	0.0
AR-/NE+	TROP2 only	21.9
AR-/NE+	Neither	78.1
AR+/NE+	Both	62.9
AR+/NE+	PSMA only	5.2
AR+/NE+	TROP2 only	21.6
AR+/NE+	Neither	10.3
AR-/NE-	Both	46.2
AR-/NE-	PSMA only	0.0
AR-/NE-	TROP2 only	50.0
AR-/NE-	Neither	3.8

TROP2-DLL3

subtype	expressed	percent
AR+/NE-	Both	0.7
AR+/NE-	TROP2 only	96.2
AR+/NE-	DLL3 only	0.3
AR+/NE-	Neither	2.7
AR-/NE+	Both	3.8
AR-/NE+	TROP2 only	1.7
AR-/NE+	DLL3 only	17.7
AR-/NE+	Neither	2.4
AR+/NE+	Both	2.4
AR+/NE+	TROP2 only	26.3
AR+/NE+	DLL3 only	3.4
AR+/NE+	Neither	1.7
AR-/NE-	Both	0.3
AR-/NE-	TROP2 only	8.2
AR-/NE-	DLL3 only	0.0
AR-/NE-	Neither	0.3

DLL3-CEACAM5

subtype	expressed	percent
AR+/NE-	Both	0.0
AR+/NE-	DLL3 only	0.3
AR+/NE-	CEACAM5 only	7.7
AR+/NE-	Neither	92.0
AR-/NE+	Both	53.5
AR-/NE+	DLL3 only	29.6
AR-/NE+	CEACAM5 only	4.2
AR-/NE+	Neither	12.7
AR+/NE+	Both	7.6
AR+/NE+	DLL3 only	4.8
AR+/NE+	CEACAM5 only	15.2
AR+/NE+	Neither	72.4
AR-/NE-	Both	0.0
AR-/NE-	DLL3 only	3.6
AR-/NE-	CEACAM5 only	53.6
AR-/NE-	Neither	42.9

PSMA-CEACAM5

subtype	expressed	percent
AR+/NE-	Both	6.8
AR+/NE-	PSMA only	70.0
AR+/NE-	CEACAM5 only	0.3
AR+/NE-	Neither	22.9
AR-/NE+	Both	0.0
AR-/NE+	PSMA only	0.0
AR-/NE+	CEACAM5 only	57.4
AR-/NE+	Neither	42.7
AR+/NE+	Both	15.5
AR+/NE+	PSMA only	55.3
AR+/NE+	CEACAM5 only	7.8
AR+/NE+	Neither	21.4
AR-/NE-	Both	42.3
AR-/NE-	PSMA only	0.0
AR-/NE-	CEACAM5 only	7.7
AR-/NE-	Neither	50.0

TROP2-CEACAM5

subtype	expressed	percent
AR+/NE-	Both	8.4
AR+/NE-	TROP2 only	88.6
AR+/NE-	CEACAM5 only	0.0
AR+/NE-	Neither	3.0
AR-/NE+	Both	14.9
AR-/NE+	TROP2 only	9.0
AR-/NE+	CEACAM5 only	43.3
AR-/NE+	Neither	32.8
AR+/NE+	Both	16.7
AR+/NE+	TROP2 only	70.8
AR+/NE+	CEACAM5 only	5.2
AR+/NE+	Neither	7.3
AR-/NE-	Both	50.0
AR-/NE-	TROP2 only	45.8
AR-/NE-	CEACAM5 only	0.0
AR-/NE-	Neither	4.2

PSMA-DLL3

subtype	expressed	percent
AR+/NE-	Both	0.0
AR+/NE-	PSMA only	76.9
AR+/NE-	DLL3 only	0.9
AR+/NE-	Neither	22.2
AR-/NE+	Both	0.0
AR-/NE+	PSMA only	0.0
AR-/NE+	DLL3 only	83.1
AR-/NE+	Neither	16.9
AR+/NE+	Both	4.5
AR+/NE+	PSMA only	60.0
AR+/NE+	DLL3 only	12.7
AR+/NE+	Neither	22.7
AR-/NE-	Both	0.0
AR-/NE-	PSMA only	48.3
AR-/NE-	DLL3 only	3.4
AR-/NE-	Neither	48.3

Supplementary Table 3. Associations between genomic alterations and TROP2, PSMA, DLL3 and CEACAM5 protein expression in the UW-TAN cohort. Estimated associations between biomarker immunohistochemistry H-score ≥ 20 and genomic calls from generalized linear mixed effects models.

**Characteristic**	**OR***	**95% CI***	**p-value**
TROP2~gAR	59.3	1.46; 2,405	0.031
TROP2~gBRCA2#	#	#	#
TROP2~gCHD1#	#	#	#
TROP2~gPTEN	1.09	0.03; 39.9	>0.9
TROP2~gRB1 BAL	0	0.00; 0.08	0.001
TROP2~gRB1 BAL_MAL	0.03	0.00; 13.8	0.3
TROP2~gTP53	1.46	0.04; 54.5	0.8
PSMA~gAR	37.2	4.41; 314	<0.001
PSMA~gBRCA2	92.7	0.94; 9,154	0.053
PSMA~gCHD1	83.2	0.27; 25,886	0.13
PSMA~gPTEN	0.46	0.05; 4.07	0.5
PSMA~gRB1 BAL	0.01	0.00; 0.09	<0.001
PSMA~gRB1 BAL_MAL	0.11	0.01; 1.90	0.13
PSMA~gTP53#	#	#	#
DLL3~gAR	0.09	0.00; 10.8	0.3
DLL3~gBRCA2#	#	#	#
DLL3~gCHD1#	#	#	#
DLL3~gPTEN	2.66	0.03; 218	0.7
DLL3~gRB1 BAL	34	0.19; 5,993	0.2
DLL3~gRB1 BAL_MAL	^	^	^
DLL3~gTP53#	#	#	#
CEACAM5~gAR	0.14	0.01; 1.39	0.094
CEACAM5~gBRCA2	#	#	#
CEACAM5~gCHD1	1.59	0.01; 276	0.9
CEACAM5~gPTEN	1.77	0.17; 18.6	0.6
CEACAM5~gRB1 BAL	4.15	0.38; 45.5	0.2
CEACAM5~gRB1 BAL_MAL	1.31	0.08; 21.1	0.9
CEACAM5~gTP53	0.45	0.04; 4.75	0.5

**OR = Odds Ratio, CI = Confidence Interval*
Regression model did not converge
^Regression model could not be estimated owing to complete separation
BAL denotes biallelic loss only
BAL_MAL includes biallelic and monoallelic loss

Supplementary Table 4. Associations between genomic alterations and *TACSTD2* , *FOLH1* , *DLL3* and CEACAM5 expression in the SU2C-WCDT. Estimated associations between biomarker expression (based on RNA-seq) and genomic calls from generalized linear mixed effects models.

**Comparison**	**OR**[1]	**95% CI**[1]	**p-value**
TACSTD2~AR	13.1	1.82; 263	0.025
TACSTD2~BRCA2	#	#	#
TACSTD2~CHD1	#	#	#
TACSTD2~PTEN	1.53	0.21; 30.7	0.7
TACSTD2~RB1	#	#	#
TACSTD2~SPOP	#	#	#
TACSTD2~TP53	1.11	0.18; 8.73	>0.9
FOLH1~AR	3.35	0.95; 11.9	0.055
FOLH1~BRCA2	#	#	#
FOLH1~CHD1	#	#	#
FOLH1~PTEN	0.72	0.21; 2.90	0.6
FOLH1~RB1	#	#	#
FOLH1~SPOP	#	#	#
FOLH1~TP53	1.04	0.31; 3.74	>0.9
DLL3~AR	0.06	0.00; 0.39	0.011
DLL3~BRCA2	2.12	0.10; 15.5	0.5
DLL3~CHD1	#	#	#
DLL3~PTEN	0.52	0.03; 3.40	0.6
DLL3~RB1	#	#	#
DLL3~SPOP	#	#	#
DLL3~TP53	2.89	0.54; 21.6	0.2
CEACAM5~AR	0.19	0.05; 0.74	0.018
CEACAM5~BRCA2	1.12	0.06; 7.24	>0.9
CEACAM5~CHD1	#	#	#
CEACAM5~PTEN	4.86	1.27; 20.6	0.022
CEACAM5~RB1	1.87	0.09; 13.4	0.6
CEACAM5~SPOP	#	#	#
CEACAM5~TP53	0.89	0.22; 3.35	0.9

[1] *OR = Odds Ratio, CI = Confidence Interval*
Regression model did not converge
