# Supplementary material for: Assessment of TROP2, CEACAM5 and DLL3 in metastatic prostate cancer: Expression landscape and molecular correlates
Source: NPJ Precis Oncol. 2024 May 17;8:104. doi: 10.1038/s41698-024-00599-6 (PMC11101486; doi:10.1038/s41698-024-00599-6)
Supplement: Supplementary file 2 — Reporting Summary [file 41698_2024_599_MOESM2_ESM.pdf]

nature portfolio

Corresponding author(s): Michael C. Haffner

Last updated by author(s): 2024/04/08

Reporting Summary

Nature Portfolio wishes to improve the reproducibility of the work that we publish. This form provides structure for consistency and transparency in reporting. For further information on Nature Portfolio policies, see our Editorial Policies and the Editorial Policy Checklist.

Statistics

For all statistical analyses, confirm that the following items are present in the figure legend, table legend, main text, or Methods section.

n/a	Confirmed	
☐	☒	The exact sample size (*n*) for each experimental group/condition, given as a discrete number and unit of measurement
☐	☒	A statement on whether measurements were taken from distinct samples or whether the same sample was measured repeatedly
☐	☒	The statistical test(s) used AND whether they are one- or two-sided<br>*Only common tests should be described solely by name; describe more complex techniques in the Methods section.*
☐	☒	A description of all covariates tested
☐	☒	A description of any assumptions or corrections, such as tests of normality and adjustment for multiple comparisons
☐	☒	A full description of the statistical parameters including central tendency (e.g. means) or other basic estimates (e.g. regression coefficient) AND variation (e.g. standard deviation) or associated estimates of uncertainty (e.g. confidence intervals)
☒	☐	For null hypothesis testing, the test statistic (e.g. *F*, *t*, *r*) with confidence intervals, effect sizes, degrees of freedom and *P* value noted<br>*Give P values as exact values whenever suitable.*
☒	☐	For Bayesian analysis, information on the choice of priors and Markov chain Monte Carlo settings
☒	☐	For hierarchical and complex designs, identification of the appropriate level for tests and full reporting of outcomes
☒	☐	Estimates of effect sizes (e.g. Cohen's *d*, Pearson's *r*), indicating how they were calculated

Our web collection on statistics for biologists contains articles on many of the points above.

Software and code

Policy information about availability of computer code

Data collection	MuTect 2 (GATK version 4.1.8.1), Strelka 2 (version 2.9.2) and VarScan 2 (version 2.4.4) SvABA, ANNOVAR (release 20200607), TitanCNA version 1.23.1 GenomicRanges version 1.38.0.
Data analysis	R package Bootstrap

For manuscripts utilizing custom algorithms or software that are central to the research but not yet described in published literature, software must be made available to editors and reviewers. We strongly encourage code deposition in a community repository (e.g. GitHub). See the Nature Portfolio guidelines for submitting code & software for further information.

Data

Policy information about availability of data

All manuscripts must include a data availability statement. This statement should provide the following information, where applicable:

- Accession codes, unique identifiers, or web links for publicly available datasets
- A description of any restrictions on data availability
- For clinical datasets or third party data, please ensure that the statement adheres to our policy

All results associated with this study are present in the paper or supplementary materials. Transcriptomic, genomic, ChIP-seq and DNA methylation data used in this study have been published previously (Gene Expression Onmibus [GEO]: GSE205056, GSE126078, GSE147250, GSE156292, GSE156290, GSE156289, GSE161948; Database of Genotypes and Phenotypes [dbGaP] phs001648). All other code and materials used in the analyses are available upon reasonable request.

Research involving human participants, their data, or biological material

Policy information about studies with human participants or human data. See also policy information about sex, gender (identity/presentation), and sexual orientation and race, ethnicity and racism.

Reporting on sex and gender	Since this study is focused on prostate cancer, all included patients are male.
Reporting on race, ethnicity, or other socially relevant groupings	Cases from the University of Washington rapid autopsy program were included. This cohort is reflective of the catchmen area of the University of Washington/Fred Hutchinson Cancer Center.
Population characteristics	Patient characteristics are shown in supplementary table 1.
Recruitment	All patients have provided written informed consent to participate in the University of Washington rapid autopsy. This study was approved by the Institutional Review Board of the University of Washington and the Fred Hutchinson Cancer Center.
Ethics oversight	This study was approved by the Institutional Review Board of the University of Washington (protocol no. 2341).

Note that full information on the approval of the study protocol must also be provided in the manuscript.

Field-specific reporting

Please select the one below that is the best fit for your research. If you are not sure, read the appropriate sections before making your selection.

☒ Life sciences ☐ Behavioural & social sciences ☐ Ecological, evolutionary & environmental sciences

For a reference copy of the document with all sections, see nature.com/documents/nr-reporting-summary-flat.pdf

Life sciences study design

All studies must disclose on these points even when the disclosure is negative.

Sample size	52 rapid autopsy cases from the University of Washington rapid autopsy program were included.
Data exclusions	No data was excluded.
Replication	For each anatomic sites two TMA cores were analyzed. For each of the 52 rapid autopsy cases, multiple metastatic sites (median number of sites per patient 7, range 1 to 21) were included.
Randomization	This is a retrospective study. No randomization was performed.
Blinding	Pathologists who scored the TMAs were blinded to the clinical and phenotypic details.

Reporting for specific materials, systems and methods

We require information from authors about some types of materials, experimental systems and methods used in many studies. Here, indicate whether each material, system or method listed is relevant to your study. If you are not sure if a list item applies to your research, read the appropriate section before selecting a response.

Materials & experimental systems

n/a	Involved in the study	
☐	☒	Antibodies
☒	☐	Eukaryotic cell lines
☒	☐	Palaeontology and archaeology
☒	☐	Animals and other organisms
☐	☒	Clinical data
☒	☐	Dual use research of concern
☒	☐	Plants

Methods

n/a	Involved in the study	
☒	☐	ChIP-seq
☒	☐	Flow cytometry
☒	☐	MRI-based neuroimaging

Antibodies

Antibodies used	DLL3 (Ventana, Clone SP347)<br>CEACAM5 (Agilent, M7072)<br>PSMA (Agilent, M3620)

	TROP2 (Abcam, ab214488)
Validation	DLL3 (Ventana, Clone SP347) – clinical grade IVD assay.<br>CEACAM5 (Agilent, M7072) – clinical grade IVD assay.<br>PSMA (Agilent, M3620) – clinical grade IVD assay.<br>TROP2 (Abcam, ab214488) – knock out validated (see vendor's website)

Clinical data

Policy information about clinical studies

All manuscripts should comply with the ICMJE guidelines for publication of clinical research and a completed CONSORT checklist must be included with all submissions.

Clinical trial registration	NA
Study protocol	NA
Data collection	NA
Outcomes	NA

Plants

Seed stocks	NA
Novel plant genotypes	NA
Authentication	NA
